# Supplementary material for: Usefulness of Docking and Molecular Dynamics in Selecting Tumor Neoantigens to Design Personalized Cancer Vaccines: A Proof of Concept
Source: Vaccines (Basel). 2023 Jun 29;11(7):1174. doi: 10.3390/vaccines11071174 (PMC10386133; doi:10.3390/vaccines11071174)
Supplement: Supplementary file 1 [file vaccines-11-01174-s001.zip › vaccines-2320089-supplementary.pdf]

SUPPLEMENTARY MATERIAL:

Table S1: Number of total interactions and hydrophobic interactions of wild-type peptides with the corresponding HLA molecule.

Gene Associated	Sequence	# total Interactions	# Hydrophobic Interactions
AKAP6	WLIDMESLV	31	21
BCSIL	ALALARKGV	29	19
DC1	VMKFKNPPV	29	19
GCNIL1	ALLETLSLLL	24	15
GOLGA3	SLDPTTSPV	23	13
HELLS	VTNSGKFLI	23	12
MRM1	LLFGMPPCL	23	14
PGM5	QQFAVGSYV	23	11
ASTN1	KPYGLDWAEL	22	15
SLC38A1	ILAALFLGL	22	13
SMARCD3	KLFEFLVYGV	22	10
USP28	LIIPFIHLI	22	13
GNL3L	NLNRCSVPV	21	11
LAMA1	STAFDFLAV	21	10
MLL2	ALSPVIPHI	20	10
PGM5	AVGSYVYSV	20	9
SIVA1	ALCGQCVRI	20	10
SNX24	KLSHQLVLL	20	11
BCSIL	ALARKGVQL	19	10
CDK4	ARDPHSGHFV	19	14
KIF3B	FALGNVISA	15	4

Table S2: Interactions between P1 and P2 in the ASTN1 wild type

#	P1	P2	Contact Score
	Atom	Atom	
1	C	N	1

2	O	N	1
3	CA	N	1
4	C	CA	1
5	O	CA	0,997
6	C	C	0,979
7	C	O	0,95
8	O	C	0,946
9	N	N	0,943
10	CB	N	0,846
11	O	O	0,807
12	C	CB	0,78
13	CA	CA	0,698
14	CG	N	0,34
15	O	CB	0,257
16	C	CG	0,251
17	CA	O	0,228
18	CA	C	0,099
19	CB	O	0,08
20	O	CG	0,056
21	N	CA	0,034
22	CB	CA	0,024
23	CA	CB	0,012
**Accumulated contact score**			**13,33**

Table S3: Interactions between P1 and P2 in the ASTN1 neoantigen

#	P1	P2	Contact Score
	Atom	Atom	
1	C	N	1
2	O	N	1
3	C	CA	0,999
4	C	CD	0,999
5	CA	N	0,999
6	O	CA	0,997
7	CA	CD	0,992
8	C	C	0,99
9	O	C	0,985
10	C	O	0,962
11	O	O	0,925
12	O	CD	0,895
13	N	N	0,887
14	CB	N	0,87
15	C	CG	0,852
16	C	CB	0,841
17	CA	CA	0,678
18	N	CD	0,581
19	CG	O	0,496
20	CA	O	0,416

21	CB	CD	0,403
22	CG	N	0,393
23	CB	O	0,275
24	O	CB	0,242
25	CA	C	0,18
26	CA	CG	0,101
27	O	CG	0,081
28	CB	CA	0,041
29	CB	C	0,031
30	CA	CB	0,019
31	N	CA	0,014
**Accumulated contact score**			**19,14**

Table S4: Interactions between P2 and P3 in the ASTN1 neoantigen

**#**	**P2**	**P3**	**Contact Score**
	**Atom**	**Atom**	
1	C	N	1
2	O	N	1
3	CA	N	1
4	C	CA	0,999
5	O	CA	0,997
6	C	C	0,983
7	CB	N	0,982
8	O	C	0,975
9	C	O	0,895
10	N	N	0,834
11	O	O	0,812

12	C	CB	0,771
13	CA	CA	0,685
14	O	CB	0,219
15	CB	O	0,144
16	CG	N	0,124
17	CB	CA	0,1
18	CA	O	0,073
19	CA	C	0,072
20	CB	C	0,026
21	CA	CB	0,013
22	N	CA	0,011
**Accumulated contact score**			**11,72**

Table S5: Interactions between P2 and P3 in the ASTN1 wild type

**#**	**P2**	**P3**	**Contact Score**
	**Atom**	**Atom**	
1	C	N	1
2	O	N	1
3	C	CA	0,999
4	CA	N	0,999
5	O	CA	0,997
6	C	C	0,977
7	O	C	0,953
8	CB	N	0,95
9	N	N	0,836
10	C	CB	0,826
11	O	O	0,716

12	C	O	0,712
13	CA	CA	0,677
14	O	CB	0,464
15	C	CG	0,333
16	CA	C	0,059
17	CG	N	0,055
18	CB	CA	0,046
19	CD	N	0,029
20	CA	CB	0,027
21	N	CA	0,012
**Accumulated contact score**			**12,67**
